# A14 SACCHAROMYCES BOULARDII CNCM I-745 SYNERGIZES WITH THE SMALL INTESTINAL MICROBIOTA TO BOOST AHR SIGNALING IN CELIAC DISEASE

**DOI:** 10.1093/jcag/gwaf042.014

**Published:** 2026-02-13

**Authors:** K Kan, M Constante, S Rahmani, G Rueda, M Pinto-Sanchez, X Roux, P Bercik, H Galipeau, A Caminero, E F Verdu

**Affiliations:** McMaster University, Hamilton, ON, Canada; McMaster University, Hamilton, ON, Canada; McMaster University, Hamilton, ON, Canada; McMaster University, Hamilton, ON, Canada; McMaster University, Hamilton, ON, Canada; Biocodex Centre de recherche, Compiègne, Hauts-de-France, France; McMaster University, Hamilton, ON, Canada; McMaster University, Hamilton, ON, Canada; McMaster University, Hamilton, ON, Canada; McMaster University, Hamilton, ON, Canada

## Abstract

**Background:**

Impaired microbial indole production and decreased activation of the aryl hydrocarbon receptor (AhR) pathway has been reported in active coeliac disease (CeD). However, *whether* and *how* this can be targeted by specific microbial therapies is unknown.

**Aims:**

We tested whether *Saccharomyces boulardii* CNCM I-745 (*S. bou*) influences indole production and AhR activation through stimulation of microbial enzymatic activity.

**Methods:**

Transgenic NOD/DQ8 mice were immunized with partially digested gliadin and cholera toxin weekly for 3 weeks followed by gluten challenges (10 mg; alternate days/ 3 weeks). Non-sensitized mice were used as controls. Mice were treated daily with 3 g/kg of *S. bou* or water during the study. Additional immunized mice treated or not with *S. bou*, received daily gavage with AhR antagonist CH-223191 (10 mg/kg) or vehicle. Small intestinal pathology was analyzed by villus to crypt (V/C) ratios and CD3^+^ intraepithelial lymphocyte (IEL) counts. Expression levels of the AhR-activated gene *Cyp1a1* were determined by RT-qPCR. Small intestinal microbiota of NOD/DQ8 mice and CeD patients were cultured in BHI media with and without 1x10^6^  *S. bou* CFUs or 1x10^8^  *Lactobacillus reuteri* (*L. reuteri*) CFUs. Free tryptophan levels, AhR activation, and total protein degradation were assayed using a commercial kit, a transfected reporter cell line, and an enzymatic assay respectively. Resulting microbiota was assessed by 16S rRNA gene sequencing and PICRUSt-predicted functions.

**Results:**

*S. bou* increased *Cyp1a1* levels, protein degradation, mucosal tryptophan and AhR activation in gluten immunized mice, paralleled by improved V/C ratios and lower IELS *vs* no probiotic, which was inhibited by AhR antagonist. In immunized mice treated with *S. bou*, microbiota analysis revealed lower PICRUSt-predicted tryptophan 2,3-dioxygenase genes, a tryptophan degrader. Mouse duodenal microbiota cultures treated with *S. bou* had higher total protein degradation, higher tryptophan levels and AhR activation *vs* cultured with no probiotic. In duodenal microbiota cultures from CeD patients the combination of *S. bou* and *L. reuteri* increased AhR activation *vs* cultures without *S. bou*.

**Conclusions:**

*S. bou* CNCM I-745 reduced gluten immunopathology in immunized NOD/DQ8 mice through activation of the AhR pathway. *In vitro* experiments suggest *S. bou* synergizes with the microbiota to increase free tryptophan from protein sources and inhibit tryptophan degradation, leading to higher indole production and AhR activation. The results identify a specific mechanistic pathway by *S. bou* CNCM I-745 in celiac disease and should encourage clinical testing in this population.

**Funding Agencies:**

CIHRBiocodex, France

